# *Sulfatase*-2 Regulates Liver Fibrosis through the TGF-β Signaling Pathway

**DOI:** 10.3390/cancers13215279

**Published:** 2021-10-21

**Authors:** Ikuo Nakamura, Faizal Z. Asumda, Catherine D. Moser, Yoo Na N. Kang, Jin-Ping Lai, Lewis R. Roberts

**Affiliations:** 1 Division of Gastroenterology and Hepatology, College of Medicine, Mayo Clinic and Mayo Clinic Cancer Center, Rochester, MN 55905, USA; inakamur@faculty.chiba-u.jp (I.N.); Moser.Catherine@mayo.edu (C.D.M.); Kang.Yoo@mayo.edu (Y.N.N.K.); Ping.Lai@mayo.edu (J.-P.L.); 2 Department of Clinical Genomics, Mayo Clinic, Rochester, MN 55905, USA; Asumda.Faizal@mayo.edu; 3 Department of Pathology, Keimyung University School of Medicine, Daegu 42601, Korea

**Keywords:** liver fibrosis, cirrhosis, SULF2, transforming growth factor-β, hepatocellular carcinoma

## Abstract

**Simple Summary:**

Liver fibrosis and/or cirrhosis is a major risk factor for hepatocellular carcinoma. Hepatic Fibrogenesis is the result of an excessive production and deposition of extracellular matrix by hepatic myofibroblasts, which are primarily formed from hepatic stellate cells. The heparan sulfate editing enzyme *sulfatase*-2 is known to be elevated in cirrhotic liver and hepatocellular carcinoma. Our aim in this study was to delineate the mechanistic role of *sulfatase*-2 in fibrotic liver disease using mouse and in vitro cell culture models of liver fibrosis. Our data here demonstrates that mice deficient in *sulfatase*-2 have reduced liver fibrosis. We also show that *sulfatase*-2 promotes cell proliferation, cell viability, the production of collagen, migration, and activation of hepatic stellate cells. Our findings highlight *sulfatase*-2 as a potential target for therapeutic intervention geared at reversing liver fibrosis.

**Abstract:**

Transforming growth factor-β (TGF-β) activates hepatic stellate cells (HSCs), which drive liver fibrosis via the production and deposition of extracellular matrix (ECM). We aimed to elucidate the mechanistic role of *sulfatase*-2 (SULF2) in liver fibrosis. To this end, we induced liver fibrosis in wild-type (WT) and SULF2 knockout (*Sulf2*-KO) mice (6–8 weeks-old) via bile duct ligation (BDL), intraperitoneal injection of carbon tetrachloride (CCl_4_) or thioacetamide (TAA). The levels of fibrosis in the liver sections were assessed via Sirius red and Masson’s trichrome staining, immunohistochemistry and immunoblotting for α-smooth muscle actin (α-SMA) and hydroxyproline. To evaluate the interaction between TGF-β and SULF2, we transfected human HSCs with scrambled control shRNA and shRNA constructs targeting SULF2 and measured α-SMA expression following treatment with TGF-β1 ligand. We show here that knockout of SULF2 significantly decreases collagen content, as well as bands of bridging fibrosis, as demonstrated by Sirius red, Masson’s trichrome and α-SMA staining after BDL, CCl_4_ and TAA injection in *Sulf2*-KO versus WT mice. In all three models of liver fibrosis, we observed significantly lower levels of hydroxyproline in the *Sulf2*-KO mice compared to the WT mice. HSCs with reduced levels of SULF2 failed to significantly express α-SMA and collagen type I following treatment with TGF-β1. Furthermore, SULF2 co-localizes with TGFBR3 and the in vitro knockdown of SULF2 in HSCs decreases the release of TGF-β1 from TGFBR3. Together, these data suggest that SULF2 regulates liver fibrosis via the TGF-β signaling pathway. Pharmacologic inhibition of SULF2 may represent a novel therapeutic approach to improve liver fibrosis.

## 1. Introduction

Fibrosis is a critical mediator of the adverse sequelae of chronic liver disease. Viral hepatitis, alcoholic and non-alcoholic steatohepatitis are the three main etiologies of liver fibrosis [1]. Although reversible in principle [2,3,4,5], unchecked fibrosis leads to end-stage cirrhosis and hepatocellular carcinoma (HCC). Accumulation of fibrotic tissue in the hepatic parenchyma involves the aggregation of preformed fibers (collapse) and formation of new fibers (fibrogenesis) [3]. Excessive production and deposition of extracellular matrix (ECM) by hepatic myofibroblasts forms the basis for hepatic fibrogenesis [3,4,5,6,7,8,9,10]. Fate-tracing of hepatic stellate cells (HSCs) demonstrates that they give rise to 82–96% of myofibroblasts in hepatotoxic liver fibrosis [7]. HSCs are therefore considered the primary drivers of pathologic liver fibrosis irrespective of etiology and as a result, are primary targets for the development of new anti-fibrotic therapies [3,4,5,6]. HSCs undergo both apoptosis and reversion to a quiescent state during the resolution of fibrosis [3,4,5]. This results in the deactivation of myofibroblasts and shifts the balance between ECM stabilizing and degrading factors (fibrogenesis and fibrinolysis) [5]. Targeting pathways that mediate HSC-induced formation and catabolism of collagen fibers is therefore a rational therapeutic approach to the prevention of end-stage liver disease. TGF-β is the most profibrogenetic cytokine in the liver [11,12]. In HSCs, TGF-β mediates the Smad2/3-induced transcription of collagen types I and III [11,12,13,14,15,16,17,18]. Transforming growth factor β receptor type III (TGFBR3) concentrates TGF-β on the cell surface following the binding of TGF-β to the extracellular domain [19]. The intracellular domain of TGFBR3 promotes the transphosphorylation of TGFBR1 by TGFBR2 [19]. The ectodomain of TGFBR3 is thought to enhance or inhibit signaling depending on the concentration of TGF-β [19]. P144 is a synthetic peptide encompassing amino acids 730–743 from the human TGFBR3 [20]. P144 blocks the biological activity of TGF-β1 [20]. Intraperitoneal administration of P144 in a rat model of carbon tetrachloride induced liver fibrosis results in fibrosis resolution [20]. Transcription of the heparan sulfate editing enzyme *sulfatase*-2 (SULF2) is known to be up-regulated in human fibrotic and cirrhotic liver [21,22] and HCC [23,24]. Furthermore, SULF2 is significantly elevated in the serum of individuals with cirrhotic liver disease [22]. SULF2 transcripts are also up-regulated in the lungs during idiopathic pulmonary fibrosis [25]. SULF2 preferentially desulfates heparan sulfate proteoglycans (HSPGs), which serve as storage sites and co-receptors for a myriad of signaling molecules, such as TGF-β, hepatocyte growth factor (HGF), fibroblast growth factor (FGF), vascular endothelial growth factor (VEGF), interleukin 6 (IL-6), and WNTs [21,26,27,28]. In the lungs, SULF2 directly regulates TGF-β, which mediates the conversion of Type II alveolar epithelial cells to myofibroblasts [25]. Our group showed that SULF2 overexpression potentiates diethylnitrosamine-induced HCC via the formation of a GLI1-STAT3 transcriptional complex [26]. The SULF2 gene is also associated with the TGF-β1 gene in human HCC [23]. We therefore aimed to elucidate the mechanistic role of SULF2 in fibrotic liver disease by examining its relationship with TGF-β. Our studies demonstrate that mice deficient in SULF2 have reduced liver fibrosis and that the in vitro knockdown of SULF2 significantly decreases the activity of TGF-β signaling in HSCs. Our data suggest that the modulation of SULF2 is a rational therapeutic strategy for the amelioration of liver fibrosis.

## 2. Materials and Methods

### 2.1. Animals and Patient Biospecimens

SULF2 knockout (*Sulf2*-KO) mice (strain name: B6;129P2-Sulf2 ^Gt(pGT1TMpfs)1Ucd^) were obtained from the Mutant Mouse Regional Resource Center (University of California, Davis, CA, USA, stock number 0403-UCD). The mice were maintained in a temperature-controlled (22 °C), pathogen-free environment and fed a standard rodent chow diet and water ad libitum. The care and use of the animals for these studies were reviewed and approved by the Institutional Animal Care and Use Committee at the Mayo Clinic. All patients consented in writing to provide study samples to the Mayo Clinic Human Specimen Repository. Institutional approval for the use of human biospecimens in this study was obtained from the Mayo Clinic Human Specimen Repository Institutional Review Board.

### 2.2. Cell Culture

The human HSC line LX-2 (provided by Dr. Vijay H. Shah, Mayo Clinic, Rochester, MN, USA, with permission from Dr. Scott Friedman), was cultured in a serum supplemented DMEM [29]. To study the effect of SULF2 on TGF-β signaling, HSCs were cultured in a medium with 1% FBS for 24 h and stimulated with 2.5 ng/mL of TGF-β1 for 18 h.

### 2.3. SULF2 Stable Transfectant Clones

Recombinant plasmids expressing full-length SULF2 cDNA cloned into the pcDNA3.1 expression plasmid (Invitrogen, Carlsbad, CA, USA) were used as described previously [30]. Geneticin-resistant clones were isolated and expanded. Stable transfections of the LX2 cell line using plasmids expressing short hairpin RNA (shRNA) sequences targeting the SULF2 mRNA cloned into the vector pSS-H1p were also performed. The target sequences used for SULF2 shRNA constructs were shRNA-a (AAGTACGTCCACAACCACA) and shRNA-b (AATGTGACTGTCACAAAAT). Constructs containing scrambled target sequences were used as controls.

### 2.4. Chemicals and Antibodies

For immunohistochemistry (IHC), we generated a rabbit polyclonal antibody against SULF2 using a peptide from the SULF2 coding sequence (amino acids 421–444: HKRDNDKVDAQEENFLPKYQRVKD, Genbank accession number NM_018837). For immunocytochemistry, we used an α-smooth muscle actin (α-SMA) antibody (Novus, Littleton, CO, USA). Protease inhibitor cocktail set III (CALBIOCHEM, San Diego, CA, USA), PVDF membrane and 4–15% Tris HCl gel (BioRad, Richmond, CA, USA), and ECL-enhanced chemiluminescence reagents (Denville Scientific Inc., Metuchen, NJ, USA) were purchased for immunoblotting. The following antibodies were purchased for immunoblotting; β-actin (Sigma-Aldrich, St. Louis, MO, USA), α-SMA (Sigma-Aldrich, St. Louis, MO, USA), TGFBR1 (Cell signaling, Danvers, MA, USA), TGFBR2 (Cell signaling, Danvers, MA, USA), TGFBR3 (sc-74511, Santa Cruz, Santa Cruz, CA, USA), phosphorylation of smad2 and smad3 (Cell Signaling, Danvers, MA, USA), for immunofluorescence; TGFBR1 (ab51870, Abcam, Cambridge, MA, USA), TGFBR2 (ab78419, Abcam, Cambridge, MA, USA), TGFBR3 (sc-74511, Santa Cruz, Santa Cruz, CA, USA) and SULF2 (sc-134045, Santa Cruz, Santa Cruz, CA, USA), and for immunoprecipitation; TGFBR3 (sc-74511, Santa Cruz, Santa Cruz, CA, USA) and TGF-β1 (ab92486, Abcam, Cambridge, MA, USA). For real time PCR, the RNeasy Mini Kit (Qiagen, Valencia, CA, USA) and High Capacity cDNA Reverse Transcription kit (Applied Biosystems, Foster City, CA, USA) were used. The following primers for real time PCR were purchased from Applied Biosystems; hSULF2 (Hs00378697) and collagen I(α)1(Hs 00164004).

### 2.5. RNA Isolation and Real-Time RT-PCR Analysis

Total RNA was prepared from liver tissue samples using the RNeasy Mini Kit. The High Capacity cDNA Reverse Transcription kit was used to produce complementary DNA. For quantitative real-time PCR analysis, primers for SULF2 and collagen I(α)1 were used in an ABI 7900 system. Each mRNA level was normalized by comparison to 18S ribosomal RNA levels in the same samples. Standard curves were prepared from synthesized SULF2 and 18S standards [30].

### 2.6. Histology and Immunofluorescence

SULF2-positive or SULF2-negative HSCs seeded on glass cover slips were incubated in 1% FBS for 24 h, after which TGF-β1 was added. Immunocytochemistry was performed at a concentration of 1/100 with antibodies against α-SMA, TGFR1, 2, 3, phosphorylated Smad 2/3, and SULF2 [30]. Confocal images of immunostained slides were obtained using a 60× oil objective. Liver samples were stained with hematoxylin-eosin, Sirius red and Masson trichrome using standard techniques. Immunostaining for SULF2 was performed as previously described [30]. Immunostaining for α-SMA was performed as indicated by the manufacturer using antibody specific for rabbit anti-α-SMA (1/200). The red-stained collagen fibers in the Sirius red staining were quantified by digital image analysis as previously described [31].

### 2.7. Western Immunoblotting

Whole liver protein lysates were prepared in a lysis buffer (FNN0011, Invitrogen, Carlsbad, CA, USA) with a protease inhibitor cocktail prior to gel electrophoresis. Blots were probed with polyclonal or monoclonal antibodies against phos-smad2, smad2, phos-smad3, and smad3, alpha-SMA, and β-actin, and incubated with secondary antibodies at 4 °C overnight. Three independent experiments were performed.

### 2.8. Flow Cytometry

Biotinylated Fluorokine human TGF-β1 (R&D system, Minneapolis, MN, USA) was used. SULF2-positive or SULF2-negative HSCs (100,000) were pelleted and resuspended at a concentration of 4 × 10^6^ cells/mL in phosphate-buffered saline (PBS). Biotinylated TGF-β1 was added (a background control with no added TGF-β1 was used), cells were incubated on ice for 30 min, and avidin-FITC reagent was added prior to reincubation on ice for 30 min in the dark. Cells were washed twice with 1× RDF1 buffer, pelleted, and resuspended in 0.2 mL of 1× RDF1 buffer with 4% paraformaldehyde. Flow cytometry was performed with a FACScan analyzer (BD Biosciences, San Jose, CA, USA).

### 2.9. Luciferase Assay

Luciferase assay was performed as previously described [32]. Approximately 50,000 HSCs with or without knockdown of SULF2 were seeded in each well of 12-well plates. After 24 h, the transfection was performed using the FuGENE^®^ HD Transfection Reagent (Roche, Madison, WI, USA) and 0.5 mg of DNA (including normalization vector and SBE12-lux aptamer vectors) according to the manufacturer’s instructions. The normalization vector and SBE12-lux aptamer vectors were provided by Dr. Ed Leof (Mayo Clinic, Rochester, MN, USA). After 6 h, the medium was replaced with a fresh 0.2% serum medium or fresh 0.2% serum medium containing 100 pM of TGF-β1. The cells were lysed after 12 h and luciferase activity was analyzed with the Luciferase Assay System (Promega, Madison, WI, USA). Data in each experiment are presented as the mean ± standard deviation of triplicates from one representative experiment.

### 2.10. Immunoprecipitation

Using an anti-TGFBR3 antibody, SULF2-positive or SULF2-negative HSCs in 10-cm dishes were washed twice with ice-cold PBS and lysed on ice for 10 min in 1ml of RIPA lysis buffer with a protease inhibitor. Pelleted cellular debris was centrifuged at 10,000 g for 10 min. The supernatant was incubated with 1 ug of mouse IgG and added to a 20 uL resuspended volume of Protein A/G PLUS-Agarose Sepharose (Santa Cruz, Santa Cruz, CA, USA). The lysate was incubated on ice for 30 min. The resultant beads were pelleted by centrifugation at 25,000 rpm for 5 min at 4 °C. The lysate was incubated with 10 uL of a mouse anti-TGFBR3 antibody on ice for 1 h and TGFBR3 proteins was immunoprecipitated by Protein A/G PLUS-Agarose Sepharose (20 μL) overnight at 4 °C. Immune complexes were pelleted by centrifugation for 5 min at 1000 g at 4 °C, washed 4 times with an RIPA buffer, and released from the beads by 3 min of boiling in 40 μL of a 1× sample buffer. 20 μL of the sample was analyzed by SDS-PAGE gel electrophoresis. Western immunoblot analysis was performed with anti TGF-β1 and anti TGFBR3 antibodies.

### 2.11. Animal Models for Liver Fibrosis

Liver fibrosis was induced by (1) Bile duct ligation (BDL); (2) chronic carbon tetrachloride (CCl4); and (3) thioacetamide (TAA) administration. For each group (BDL, CCl4, TAA) we used 10 WT and 10 Sulf2-KO mice (*n* = 10). For all procedures, the mice were kept in a 2 L induction chamber (1.5% isoflurane and 1 L/min O_2_) until loss of pedal reflex and transferred to the nose cone. Mice were monitored for behavioral or clinical signs of pain 3–5 times a week during the study period. Euthanasia was achieved by slowly exposing mice to increasing levels of CO_2_ delivered to a micro-isolator. Death was verified by cessation of respiratory and cardiovascular movements by observation at room air for at least 10 min.

(1) Bile duct ligation (BDL): Bile duct ligation was performed as described previously [32]. Briefly, cohorts of Sulf2-KO and WT male littermates which were 6–8 weeks old were subjected to BDL. At 14 or 21 days after BDL, the animals were sacrificed to estimate liver fibrosis.

(2) Chronic carbon tetrachloride (CCl_4_) administration: WT and Sulf2-KO mice at 4–6 weeks of age were treated twice a week with 8 or 16 consecutive intraperitoneal (i.p.) injections of 0.5 mL/kg CCl_4_(dilute 1; 10) to induce liver fibrosis. The animals were sacrificed 4 or 8 weeks post injections to estimate liver fibrosis.

(3) Thioacetamide (TAA) administration: WT and Sulf2-KO mice at 4–6weeks of age were treated 3 times a week with 18 consecutive intraperitoneal (i.p.) injections of 150 mL/kg TAA. The animals were sacrificed at 6 weeks post injections to estimate liver fibrosis. To measure serum ALT, the mice were anesthetized and blood was collected at 3 days after BDL or 24 h after the first injection of CCl_4_ or TAA. Liver tissue was frozen in liquid nitrogen for analysis. Liver samples were also fixed in 10% formalin, embedded in paraffin, and stained with hematoxylin-eosin (H&E) for histological analysis.

### 2.12. Clinical Information on Patients with or without Cirrhosis

Tumor and adjacent benign tissues from 33 individual HCC patients undergoing surgical resection for HCC were obtained from the Mayo Clinic in Rochester, MN, USA, Resected liver tissue was frozen in liquid nitrogen for analysis. Liver samples were fixed in 10% formalin, embedded in paraffin, and stained with hematoxylin-eosin (H&E) and SULF2 antibody for histological analysis. The clinical and pathological features for the 33 individual liver samples are presented in Supplementary Table S1. The median age of the individuals was 64.2; 48.5% were male. In 12 samples with non-cirrhosis and 10 samples with cirrhosis, immunohistochemistry for SULF2 was performed.

### 2.13. Migration Assay

Migration assay was performed as described previously [23]. SULF2-positive or SULF2-negative LX2 cells were plated onto 6-well plates and grown to confluency. Wounds were induced with a 200 μL pipette tip. The wounds were photographed with a phase-contrast microscope at 0 and 12 h. Cell migration was quantitated by measuring the width of each wound. The experiments were repeated 3 times.

### 2.14. Hydroxyproline Content

Total collagen was determined by hydroxyproline quantification as previously described [33], with minor modifications. Briefly, mouse liver tissue was hydrolyzed with 6N HCl at 110 °C for 16 h. Samples and an hydroxyproline standard were incubated with chloramine-Tbuffer for 20 min at room temperature. Ehrlich’s reagent was added and the samples were again incubated for 15 min at 65 °C. Absorbance of each sample was measured at 550 nm using a microplate reader (Packard BioScience, Meriden, CT, USA). Hydroxyproline levels were calculated against standard curves of 4-hydroxy-L-proline (Sigma-Aldrich, St. Louis, MO, USA) and expressed as mg hydroxyproline per gram of liver tissue.

### 2.15. Scoring of Immunohistochemistry

The degree of staining intensity (0, none; 1, weak; 2, moderate; 3, strong) and the proportion of positive hepatocytes in non-tumorous liver parenchyma (0, 0–10%; 1, 10–25%; 2, 25–50%; 3, 50–100%) were manually scored in samples stained for SULF-2 (12 non-cirrhosis and 10 cirrhosis samples). SULF-2 was positive if the cytoplasm of 10% or more hepatocytes were stained by using a representative rabbit polyclonal antibody. The SULF-2 combined score in each sample was calculated based on the sum of the scores for staining intensity and proportion of positive hepatocytes in non-tumorous liver parenchyma.

### 2.16. Statistical Analysis

All data represent at least 5 (maximum of ten) independent mice and are expressed as the mean ± SEM. Differences between groups were compared using a two-tailed Student’s *t*-test (* *p* < 0.05, ** *p* < 0.01).

## 3. Results and Discussion

### 3.1. Expression Levels of SULF2 in Fibrotic and Non-Fibrotic Liver

**Figure 1:**
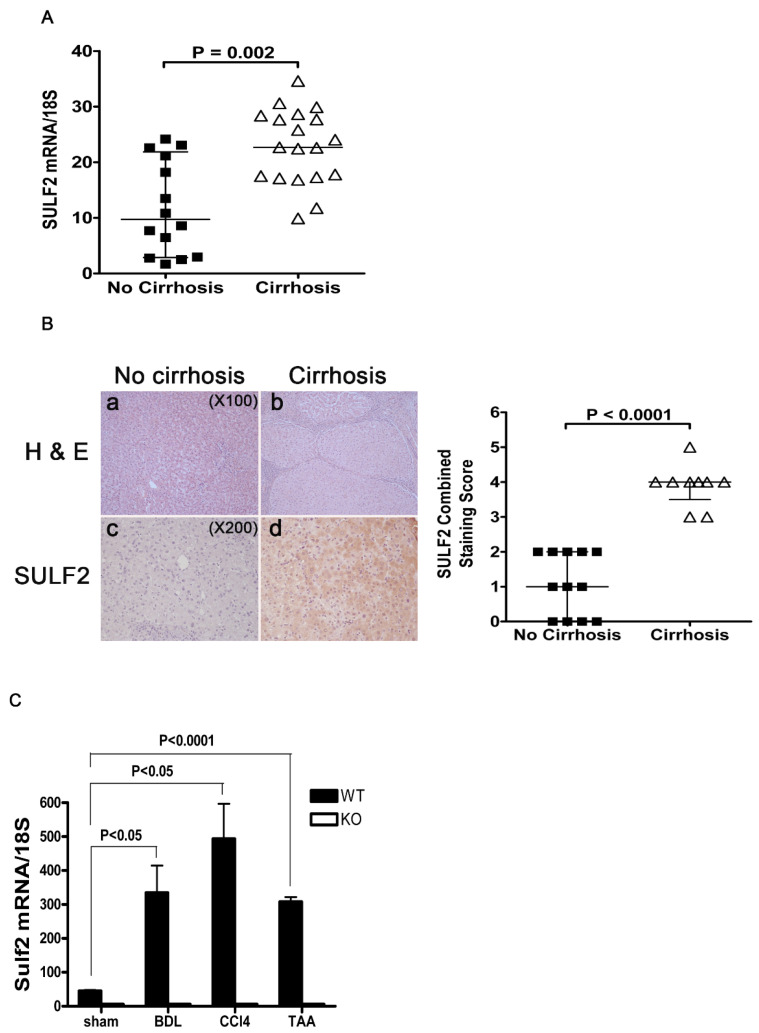
Expression of SULF2 in cirrhotic patients and mice with liver fibrosis. (**A**) The levels of SULF2 mRNA in liver samples Figure 1. and non-cirrhotic (*n* = 14) patients. (**B**) Few inflammatory cells are observed in the portal tract without fibrotic changes (H&E, ×100) (a). The micronodular cirrhotic nodules are surrounded by thick fibrous tissue bands and numerous inflammatory cells (H&E, ×100) (b). The expression of SULF2 protein in the liver parenchyma adjacent to HCC is depicted in (c,d). All hepatocytes in non-cirrhotic liver parenchyma adjacent to HCC show no staining by sulf-2 antibody (c). Most hepatocytes in cirrhotic liver parenchyma adjacent to HCC are stained with sulf-2 antibody to varying degrees. The adjacent graph shows combined SULF2 staining scores in non-cirrhotic liver (*n* = 12) and cirrhotic liver (*n* = 10). (**C**) RT-PCR of SULF2 mRNA levels in mouse livers. Mice were sacrificed at 21 days after bile duct ligation (BDL), 8 weeks after treatment with carbon tetrachloride (CCL_4_) and 6 weeks after treatment with thioacetamide (TAA). Data shown are representative of ten mice per genotype per time point and are presented as mean ± SEM.

SULF2 is known to be increased in the serum of cirrhotic liver and HCC patients compared to non-cirrhotic liver [21,22,23,24]. We therefore assessed SULF2 transcripts and protein expression levels in the specimens of cirrhotic and non-cirrhotic patients. Gene expression levels of SULF2 were significantly up-regulated in cirrhotic compared to non-cirrhotic liver (Figure 1A). We compared liver specimens from patients with concomitant HCC and surrounding cirrhotic tissue to patients with HCC without cirrhosis. Immunohistochemical staining showed SULF2 protein expression in the liver parenchyma adjacent to HCC (Figure 1B). Most hepatocytes in cirrhotic liver parenchyma adjacent to HCC were positive for SULF2 (Figure 1B). In comparison, hepatocytes located in non-cirrhotic liver parenchyma adjacent to HCC showed no positivity for SULF2 (Figure 1B). The staining score in cirrhotic liver specimens was significantly higher than that of non-cirrhotic specimens (Figure 1B). We further assessed the association between SULF2 levels and fibrotic liver via three different mouse models of fibrotic liver (bile duct ligation (BDL), carbon tetrachloride (CCl_4_), and thioacetamide (TAA) treatment). The levels of SULF2 mRNA in the livers of WT mice following BDL, treatment with CCl_4_ and TAA are significantly higher than in the livers of non-treated mice (Figure 1C).

### 3.2. Knockout of SULF2 Suppresses Liver Fibrosis

**Figure 2:**
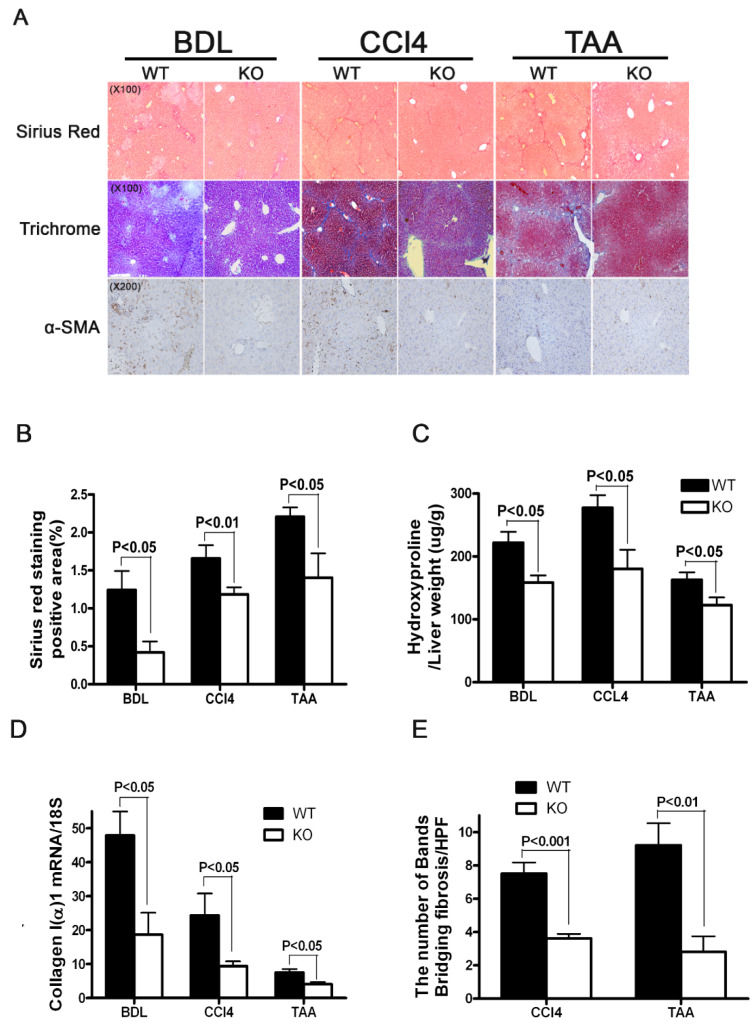
Liver fibrosis is decreased in SULF2-KO mice. (**A**) Histological analysis of livers from WT and SULF2-KO mice shows hepatic fibrosis and HSC activation. Livers were harvested, fixed in formalin, and stained for collagen content with Sirius red and Masson trichrome and for the analysis of HSC activation with α-SMA. (**B**) Positive areas stained with Sirius red were quantitated using digital image analysis. Representative photomicrographs of liver sections are depicted (100× magnification). (**C**) Collagen content of WT and SULF2-KO mouse livers. (**D**) The mRNA levels of collagen I(α)1 in mice with liver fibrosis. (**E**) The number of bridging fibrosis in Sirius red staining. Bridging fibrosis was counted under 100× magnification. Mice were sacrificed 21 days after bile duct ligation (BDL), 8 weeks after treatment with carbon tetrachloride (CCL_4_) and 6 weeks after treatment with thioacetamide (TAA). Data shown are representative of ten mice per group and are presented as mean ± SEM.

To further investigate the role of SULF2 in liver fibrosis, we induced fibrosis in *sulf2*-KO mice via a BDL, CCl_4_ and TAA treatment. Staining with Sirius red, Trichrome staining of α-SMA and hydroxyproline levels were determined. In addition, the expression levels of collagen 1(α)I mRNA was determined. The number of bands of bridging fibrosis in tissues stained with Sirius red were quantified. We observed significantly lower levels of Sirius red staining from fibrotic liver tissues (BDL, CCl4 and TAA) in *sulf2*-KO than WT specimens (Figure 2A,B). Furthermore, the positive area of trichrome and α-SMA staining in WT mice was larger than in *sulf2*-KO mice (Figure 1A). The levels of hydroxyproline in *sulf2*-KO mice were lower than WT mice in all three different models of fibrotic liver disease (Figure 2C). The expression level of collagen 1(α)I transcripts in *sulf2*-KO mice was significantly lower than that of WT mice (Figure 2D). Similarly, we observed significantly lower levels of bridging fibrosis in *sulf2*-KO mice compared to WT mice following the CCl_4_ and TAA treatment, (Figure 2E).

### 3.3. The Effect of SULF2 on Liver Injury

**Figure 3:**
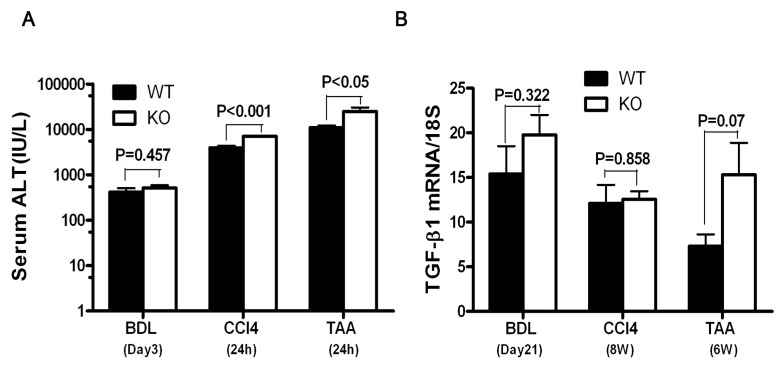
SULF2 affects activation of the TGF signaling pathway in mice liver fibrosis. (**A**) Serum ALT levels in mice with liver fibrosis. (**B**) TGF-β1 transcript levels in fibrotic mice liver tissue. Data shown are representative of ten mice per group and are presented as mean ± SEM.

To determine whether SULF2 affects acute liver injury after BDL or the administration of CCL_4_ or TAA, we measured serum ALT levels. At 3 days post BDL, serum ALT was nominally increased in *sulf2*-KO mice compared to WT mice (*p* = 0.457, Figure 3A). On the other hand, after 24 h of CCL_4_ and TAA treatment, the levels of serum ALT in *sulf2*-KO were significantly higher than in WT mice (*p* < 0.001, *p* < 0.05, Figure 3A), suggesting that *sulf2*-KO mice are more prone to toxin-induced injury.

### 3.4. The Effect of SULF2 on the Activation of TGF-β1 Signaling and α-SMA after Chronic Liver Injury

To elucidate the mechanistic role of SULF2 in chronic liver injury, we first investigated whether SULF2 directly increased the production of TGF-β1 following chronic liver injury. We measured the expression levels of TGF-β1 mRNA following the BDL, CCl_4_ and TAA treatment by RT-PCR. We did not detect a significant difference in the expression levels of TGF-β1 transcripts in the fibrotic liver specimens of WT and *sulf2*-KO mice (Figure 3B). This data suggests that SULF2 has no direct effect on the up-regulation of TGF-β1 mRNA following chronic liver injury. TGF-β1 expression is known to be consistently elevated in fibrotic tissue [13,14,15]. Furthermore, SMAD proteins are known to act as transcription effectors of TGF-β1 [13,14,15]. We therefore hypothesized that SULF2 modulates the activation of TGF-β1 in chronic liver injury via a SMAD dependent pathway. Our group has previously shown that the extracellular matrix protein periostin (POSTN) is an effector protein in SULF2-induced angiogenesis in hepatocellular carcinoma tumors [34]. We identified the TGF-β1/SMAD pathway as a critical signaling axis between SULF2 and upregulation of POSTN transcription [34]. Our previously published data [34] showed that expression of SULF2 in human hepatoma Hep3B cells results in activation of TGF-β1 downstream signaling via upregulation of phospho-SMAD2 and phospho-SMAD3 expression. Taken together, these results show that while SULF2 does not directly up-regulate the transcription of TGF-β1 mRNA, it modulates TGF-β1 via activation of the TGF-β1-Smad2/3 signaling pathway following chronic liver injury.

### 3.5. The Effect of SULF2 Knockdown on Human Hepatic Stellate Cells

**Figure 4:**
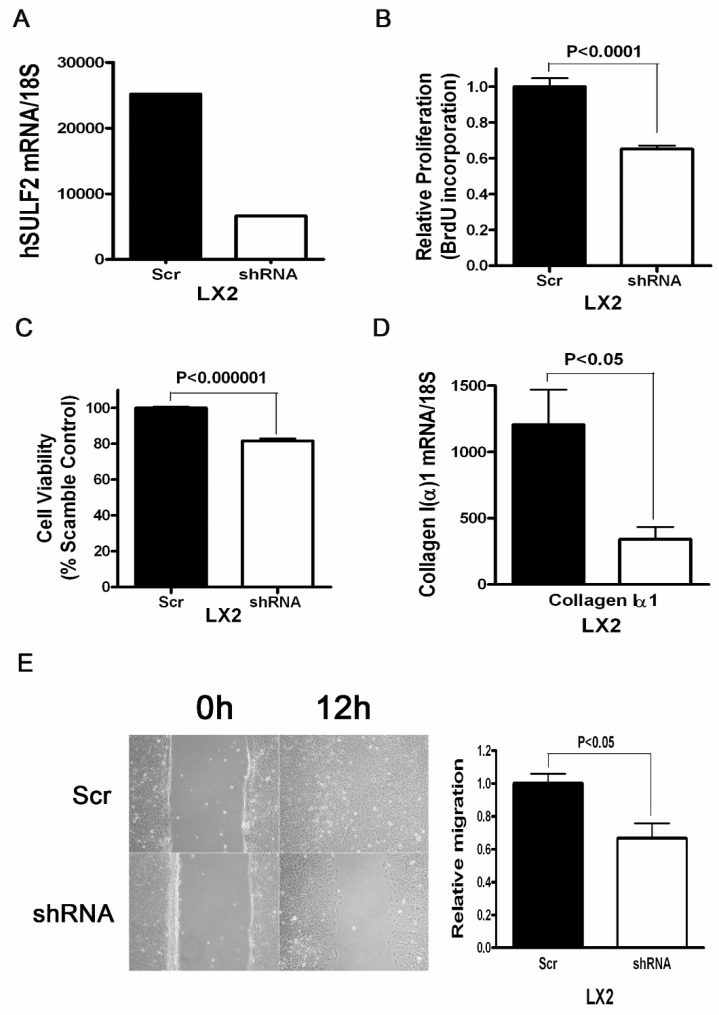
The effect of SULF2 on human hepatic stellate cells. (**A**) Plasmid constructs expressing an shRNA targeting the SULF2 mRNA or the empty vector (scrambled shRNA) plasmid pSS-H1p were stably transfected into human hepatic stellate cells (HSCs), which express high levels of SULF2 mRNA. SULF2 mRNA levels were quantitated by real-time RT-PCR in HSC scrambled and the SULF2 shRNA clone. SULF2 mRNA levels were significantly suppressed by the shRNA plasmid. (**B**) BrdU incorporation in HSCs transfected with Scr vs. SULF2 shRNA. (**C**) MTT assay in HSCs transfected with Scr vs. SULF2 shRNA. (**D**) The levels of collagen I(α)1 mRNA in HSCs. (**E**) Scratch wounds were induced in confluent cell culture monolayers with a 200 μL pipette tip; photomicrographs of the wounds were taken at 0 and 12 h post induction. Relative migration of the wound edge was quantitated. We observed a significantly decreased rate of migration in HSC transfected with shRNA relative to HSC transfected with the scrambled shRNA plasmid. Data in each experiment are presented as the mean ± standard deviation of triplicates from one representative experiment.

To explore the role of SULF2 on human hepatic stellate cell activation, we performed a BrdU assay for cell proliferation, a wound healing assay for invasive ability and an MTT assay for cell viability. We also assessed the levels of collagen 1(α)I production in SULF2 deficient HCSs compared to wild-type HSCs. SULF2 was stably knocked down in HSCs using a plasmid construct expressing an shRNA targeting the SULF2 mRNA (Figure 4A). Knockdown of SULF2 significantly decreased the proliferation and viability of HSCs (Figure 4B,C). The production of collagen I(α)1 in SULF2 deficient HSCs was significantly lower than in wild-type HSCs (Figure 4D). The wound healing assay showed that knockdown of SULF2 decreases the invasive ability of HSCs (Figure 4E).

### 3.6. In Vitro Knockdown of SULF2 Inhibits the TGF-β Signaling Pathway in Human Hepatic Stellate Cells

**Figure 5:**
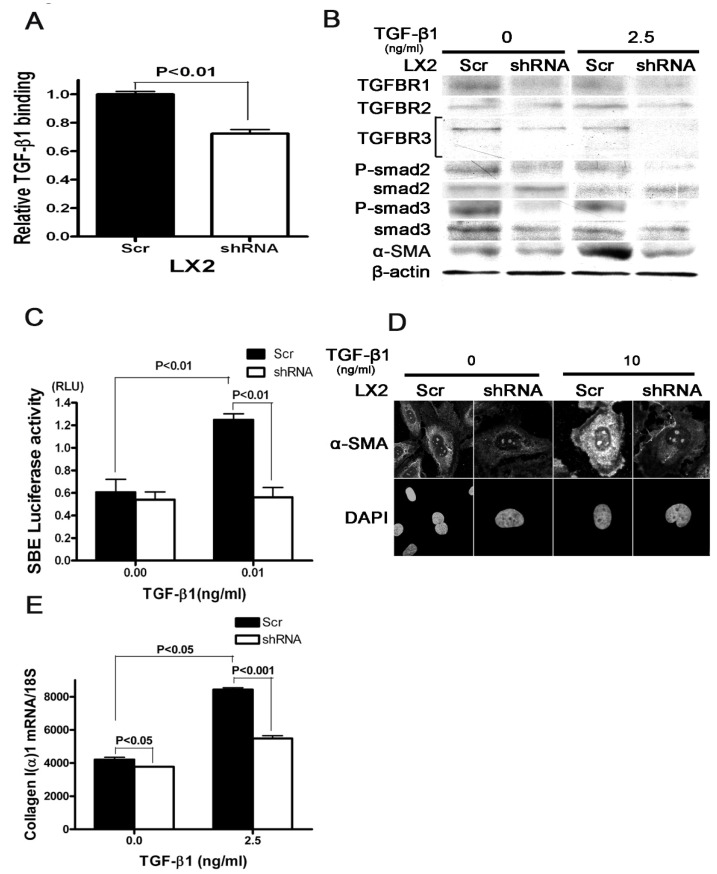
SULF2 knockdown reduces the activation of the TGF-β signaling pathway after TGF-β1 stimulation in human hepatic stellate cells. (**A**) Relative TGF-β1 binding in human hepatic stellate cells by flow cytometry. (**B**) Expression of TGF-β receptors, phosphorylation of smad2/3 and α-SMA at 24 h after TGF-β1 stimulation. (**C**) Luciferase assay at 12 h after TGF-β1 stimulation using the normalization vector and SBE12-lux aptamer vectors. (**D**) Expression of α-SMA in human HSCs at 24 h after TGF-β1 stimulation in immunofluorescence (20×). (**E**) Levels of collagen I(α)1 mRNA in human HSCs at 24 h after TGF-β1 stimulation by RT-PCR. (**B**,**D**,**E**) Cells were incubated in 1% FBS for 24 h and then TGF-β1 (2.5 ng/mL) was added. After 24 h, the assay was performed. 18S is shown as loading controls. Data in each experiment are presented as the mean ± standard deviation of triplicates from one representative experiment. Uncropped Western Blot figures are shown in Figure S1.

**Figure 6:**
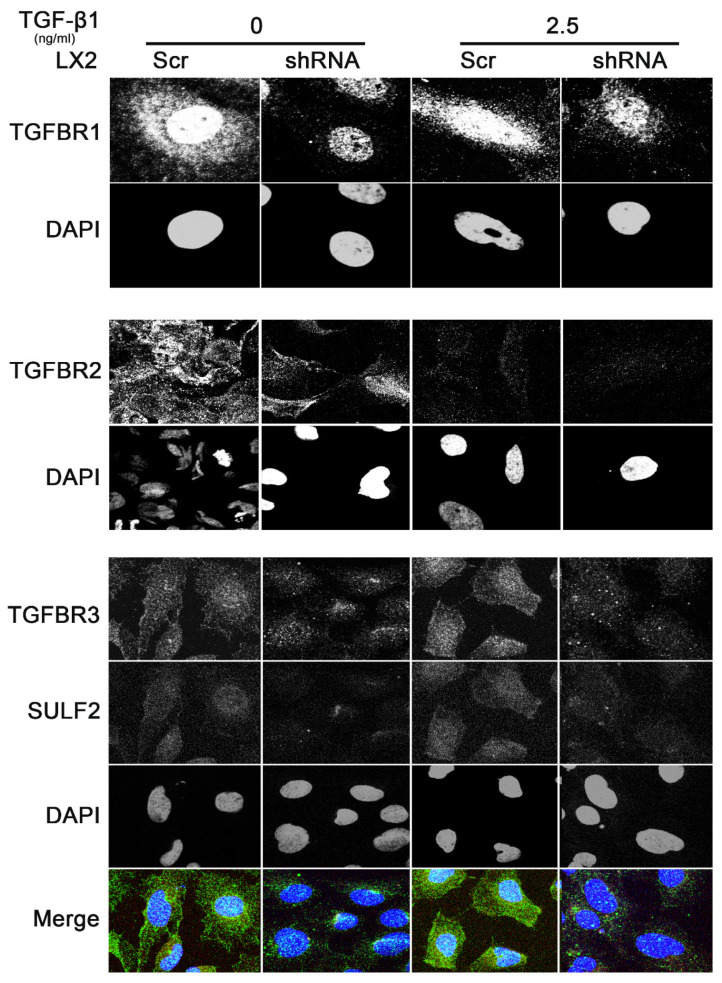
Immunofluorescence analysis of the effect of SULF2 on TGF-β receptors in human hepatic stellate cells (20×). The expression of TGFBR1 (top row in panel 1); TGFBR2 (top row in panel 2); TGFBR3 and SULF2 (top and bottom row, respectively in panel 3) after TGF-β1 treatment. SULF2 decreased the expression of TGFBR1, 2 and 3 and co-localized with TGFBR3. HSCs were incubated in 1% FBS for 24 h prior to the addition of TGF-β1 (2.5 ng/mL). Experiments were performed in triplicate. Representative micrographs are shown.

TGFBR2 and TGFBR3 are both related transmembrane serine/threonine kinase receptors which hetero-oligomerize to transduce signaling [35]. The basic mechanism involves the binding of TGF-β superfamily ligands to the type II receptor, which in turn catalyzes the phosphorylation of the type I receptor [35]. TGFBR3, also known as betaglycan, is a broadly distributed heparan and chondroitin sulfate proteoglycan [35]. TGFBR3 is the most abundant of the TGFB receptors yet; it enhances the binding of ligands to the TGFBRI and TGFBR2 signaling complexes [35]. The ectodomain of TGFBR3 enhances or inhibits signaling depending on the concentration of TGF-β [19]. Our data suggest that SULF2 modulates TGF-β1 via activation of the TGF-β1-Smad2/3 signaling pathway following chronic liver injury. We conducted flow cytometry analysis to assess the effect of SULF2 on the in vitro binding of TGF-β1 in HSCs by using a biotinylated Fluorokine human TGF-β1. Knockdown of SULF2 decreased the relative binding of TGF-β in HSCs (Figure 5A). We observed significantly lower levels of TGFBR1 and 2 expression in SULF2 deficient compared to wild-type HSCs following stimulation with TGF-β1 (Figure 5B or Figure 6). The phosphorylation of smad 2 and 3 was also decreased in SULF2 deficient HSCs (Figure 5B). To determine whether SULF2 inhibits the activation of the TGF-β signaling pathway, we conducted a luciferase assay using the SBE12-lux aptamer vector. We observed significantly lower levels of luciferase activity in SULF deficient HSCs following TGF-β1 stimulation (Figure 5C). Based on these data, we sought to establish a direct role for SULF2 in the activation of HSCs. To achieve this, we conducted immunoblotting and immunofluorescence for α-SMA in SULF2 deficient and wild-type HSCs following stimulation with TGF-β1. The expression of α-SMA in wild- type HSCs increased significantly following TGF-β1 stimulation (Figure 5B,D). On the other hand, knockdown of SULF2 significantly decreased the expression of α-SMA with or without TGF-β1 stimulation. Furthermore, stimulation with TGF-β1 results in a decreased production of collagen I(α)1 SULF2 deficient HSCs (Figure 5E).

### 3.7. SULF2 Co-Localizes with TGFBR3 and TGF-β1 Forms a Complex with TGFBR3

**Figure 7:**
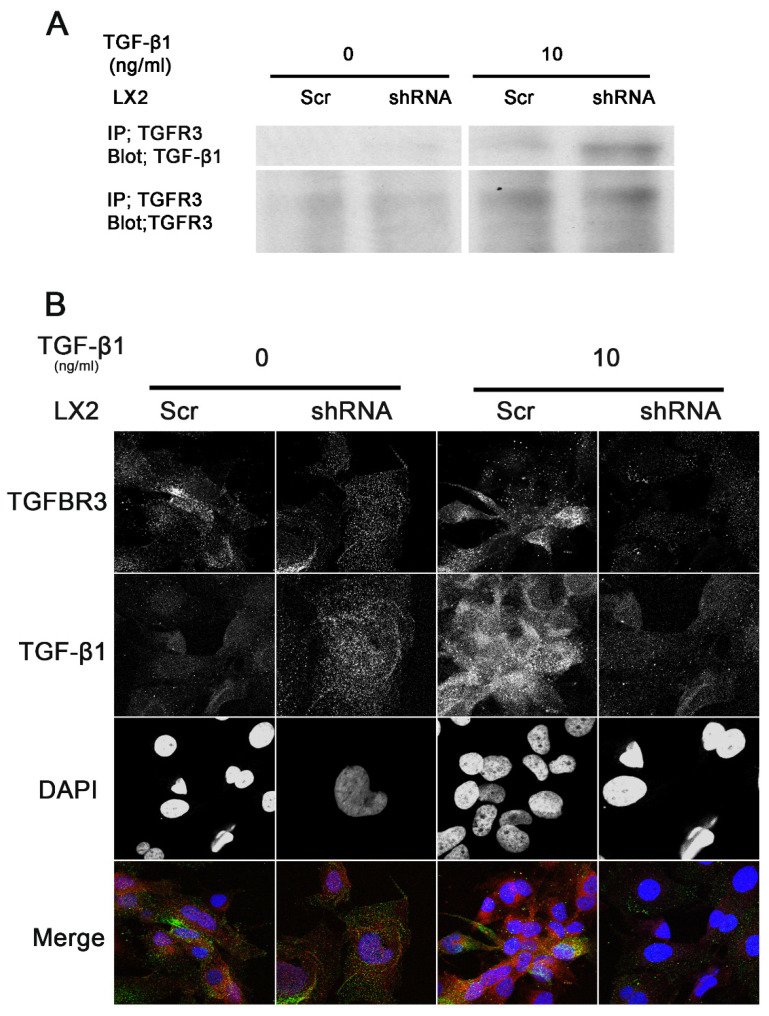
SULF2 modulates the release of TGF-β1 from TGFBR3 (20×). (**A**) Immunoprecipitation shows that TGF-β1 forms a complex with TGFBR3 and that SULF2 regulates this complex. (**B**) Immunofluorescence shows that SULF2 modulates the release of TGF-β1 from TGFBR3. HSCs were incubated in 1% FBS for 24 h prior to the addition of TGF-β1 (2.5 ng/mL). Experiments were performed in triplicate. Representative micrographs are shown. Uncropped Western Blot figures are shown in Figure S1.

We previously demonstrated that SULF2 co-localizes with the heparan sulfate proteoglycan Glypican-3 [35]. We therefore hypothesized that SULF2 co-localizes with betaglycan (TGFBR3). To determine whether SULF2 co-localizes with TGFBR3 we conducted double immunofluorescence staining of TGFBR3 and SULF2. We observed co-localization between SULF2 and TGFBR3 (Figure 6). To explore this phenomenon further, we examined the ability of TGF-β1 to form a complex with TGFBR3 in HCC cells. To this end, we conducted immunoprecipitation and double immunofluorescence with the TGF-β1 and TGFBR3 antibodies. As shown in Figure 7A,B, TGF-β1 forms a complex with TGFBR3. Furthermore, the knockdown of SULF2 results in an increased expression of the TGF-β1-TGFBR3 complex (Figure 7A). TGFBR3 is thought to classically function primarily by presenting the TGF-β ligand to the type II receptor. Our data suggest that there might be a larger role for the type III receptor in TGF-β signaling. Others have shown that cells that do not express the type III receptor, express the closely related receptor endoglin, which shares ~70% homology with the type III receptor in the cytoplasmic domain [33,34,35,36,37,38]. It has also been established that these type III receptor deficient cells respond to TGF-β1 but are unresponsive to TGF-β2 [34]. By ectopically expressing TGFBR3 in these cells, it is possible to restore sensitivity to TGF-β2 [34]. We show here that the TGF-β1-TGFBR3 complex is modulated by SULF2 (Figure 7A). This suggests that the TGFBR3 likely plays an important role in the SULF2 mediated activation of the TGF-β1 signaling pathway.

## 4. Discussion

The hallmark of fibrotic disease is the increased production of type I collagen. Progression to HCC occurs within the setting of a liver microenvironment that consists of fibrogenesis and cirrhosis. The dynamic balance between collagen production and degradation is controlled by several growth factors and cytokines, of which TGF-β is the most important. Our group and others have shown that the heparan sulfate editing enzyme SULF2 is up-regulated in human fibrotic and cirrhotic liver [21,22] and HCC [23,24,25,26,30,34,38].

Heparan sulfate proteoglycans are co-receptors for several heparin-binding growth factors and cytokines and are critical for cell signaling. Heparan sulfate 6-*O*-endosulfatases, such as SULF2, act as endosulfatases to remove 6-*O*-sulfate groups from heparan sulfate. This results in downstream effects such as the alteration of binding sites for signaling molecules [24,25,26,27,39]. In this study, we showed that the expression of SULF2 is up-regulated in cirrhotic human liver, as well as in fibrotic mouse livers. This suggests that SULF2 plays a role in both fibrosis and subsequent tumorigenesis. This is consistent with our previous findings that the transcriptional induction of Periostin by the SULF2-TGF-β1-SMAD axis drives angiogenesis in HCC [34,38]. SULF2 overexpression is also known to potentiate diethylnitrosamine-induced HCC [26]. SULF2 is known to mediate the release of several signaling factors, including ligands of Hedgehog, WNT and TGF-β pathways [26].

TGF-β expression is markedly increased in the cirrhotic liver [39,40,41]. SULF2 has been shown to be closely related to TGF-β signaling in human HCC [38,42]. Our data in this study suggest that SULF2 does not directly increase the expression levels of TGF-β1 mRNA following chronic liver injury. Instead, SULF2 modulates the phosphorylation of smad2 and 3 downstream of TGF-β1 signaling in liver fibrosis induced by BDL and treatment with CCl_4_ and TAA. This mechanistic role of SULF2 is manifested in the amelioration of liver fibrosis in mice following the knockout of SULF2. TGF-β is a potent inducer of stellate cell proliferation and collagen production [11,12,13,14,15,16,17,18,19,41,43]. In this study, we demonstrated that in human hepatic stellate cells, the knockout of SULF2 inhibits cell proliferation, cell viability, the production of collagen I(α)1, migration and activation. We further showed by stimulation of HSCs with TGF-β1 that SULF2 regulates the activation of the TGF-β signaling pathway. TGFBR3 is known to concentrate TGF-β on the cell surface following the binding of TGF-β to the extracellular domain [43,44,45]. Our data in this study show that SULF2 modulates the expression of TGFBR3 and the binding of TGF-β is decreased in SULF2 deficient HSCs following treatment with TGF-β1. This is consistent with our observation that SULF2 modulates TGF-β1 via activation of the TGF-β1-Smad2/3 signaling pathway following chronic liver injury.

Several different pathways and cytokines have been shown to be involved in the progression of liver fibrosis. But the role of sulfatase 2 in liver fibrosis has not been closely examined. Our evidence here provides insight into a novel role for SULF2 in liver fibrosis. We demonstrate here that SULF2 exerts its effect on fibrotic liver disease via its interplay with the TGF-β1/Smad pathway and that the TGFBR3 likely plays an important role in the SULF2 mediated activation of the TGF-β1 signaling pathway. Our data highlight SULF2 as a suitable target for the amelioration of liver fibrosis.

## 5. Conclusions

In this study, we focused on the mechanistic role of SULF2 in fibrotic liver disease in mouse models of liver fibrosis and under in vitro conditions using HSCs. Here, we show a novel role for SULF2 in liver fibrosis. Our studies specifically demonstrate that: (1) SULF2 transcripts are highly expressed in the liver of cirrhotic patients; (2) knockout of SULF2 ameliorates liver fibrosis in mice following BDL and treatment with CCl_4_ and TAA; (3) SULF2 promotes cell proliferation, cell viability, the production of collagen I(α)1, migration, and activation of HSCs; (4) SULF2 modulates the activity of the TGF-β signaling pathway in HSCs; (5) TGF-β1 forms a complex with TGFBR3 (betaglycan).

## Data Availability

The data presented in this study are available in insert article or supplementary material here.

## Supplementary Materials

The following are available online at https://www.mdpi.com/article/10.3390/cancers13215279/s1, Figure S1: Uncropped Western Blot Figures. Table S1: Clinical and pathological features for individual liver samples.
