# Supplementary material for: Sulfatase-2 Regulates Liver Fibrosis through the TGF-β Signaling Pathway
Source: Cancers (Basel). 2021 Oct 21;13(21):5279. doi: 10.3390/cancers13215279 (PMC8582359; doi:10.3390/cancers13215279)
Supplement: Supplementary file 1 [file cancers-13-05279-s001.zip › cancers-1337354-Supplementary.pdf]

cancers

MDPI

Supplementary Materials: *Sulfatase 2* Regulates Liver Fibrosis through the TGF-β Signaling Pathway

Ikuo Nakamura, Faizal Z. Asumda, Catherine D. Moser, Yoo Na N. Kang, Jin-Ping Lai and Lewis R. Roberts

(A)

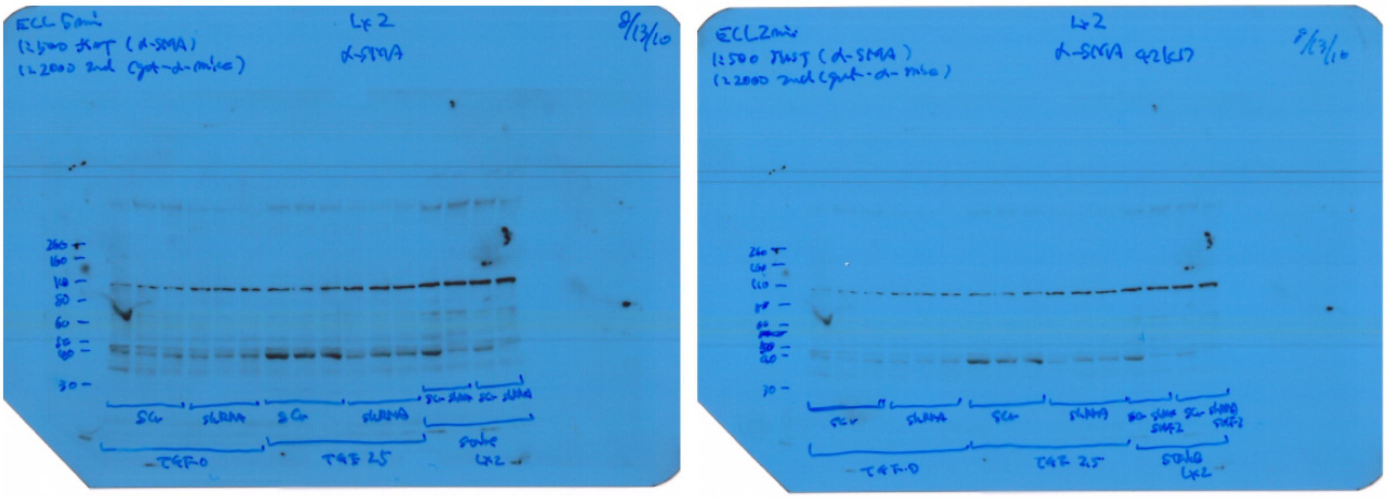

(**B**)

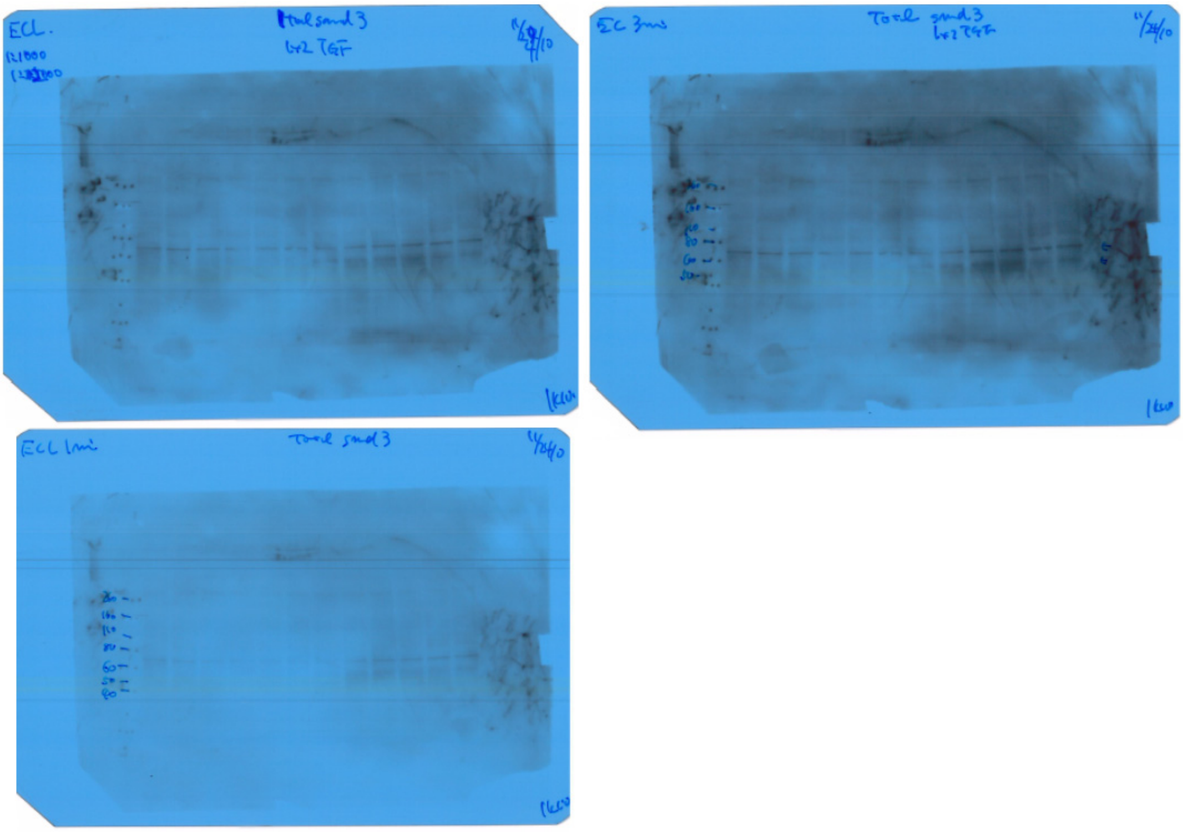

(**C**)

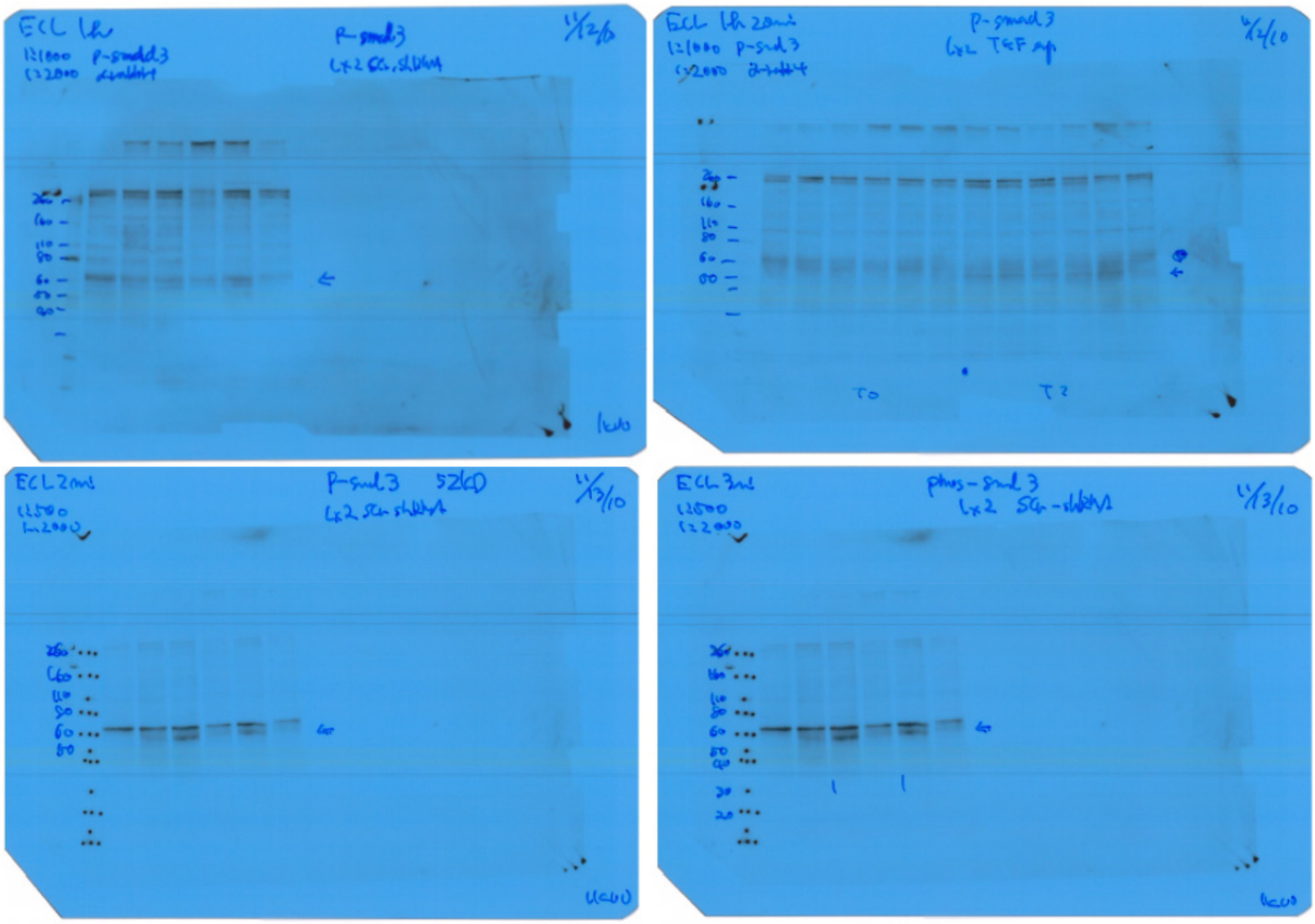

(D)

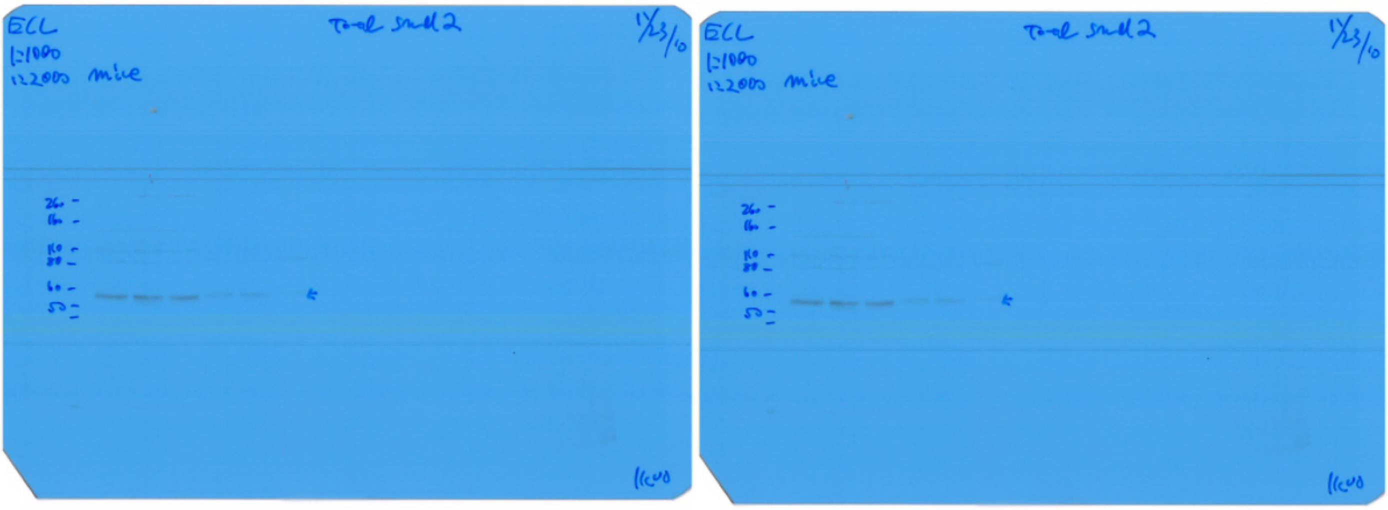

(E)

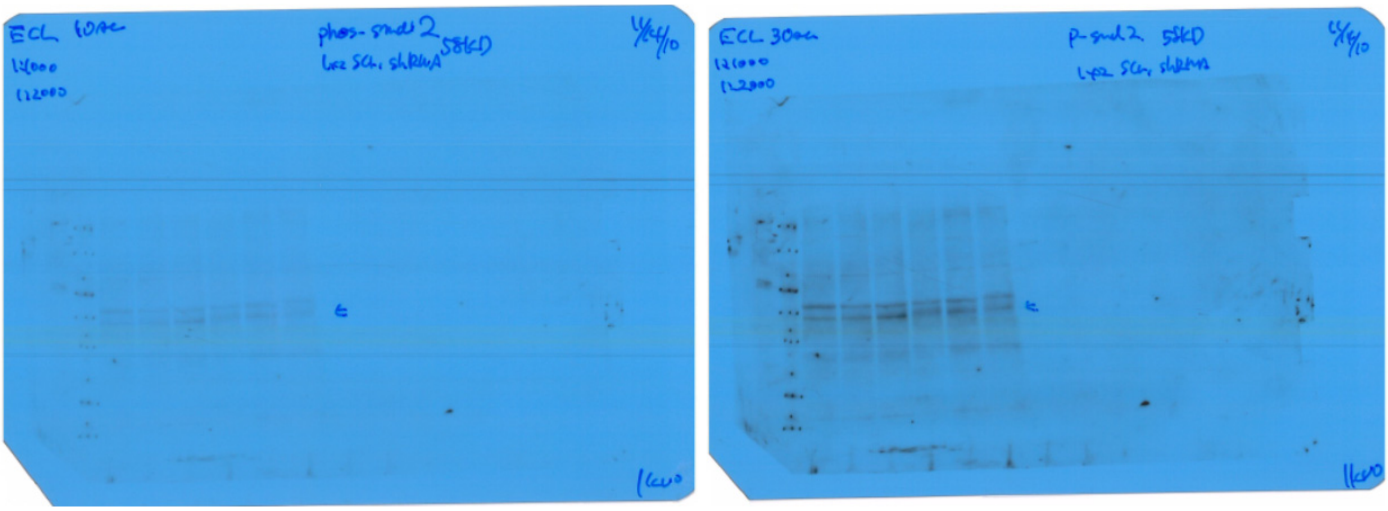
ECL
phos-smad2 55KD
ECL
p-smad2 55KD

(**F**)

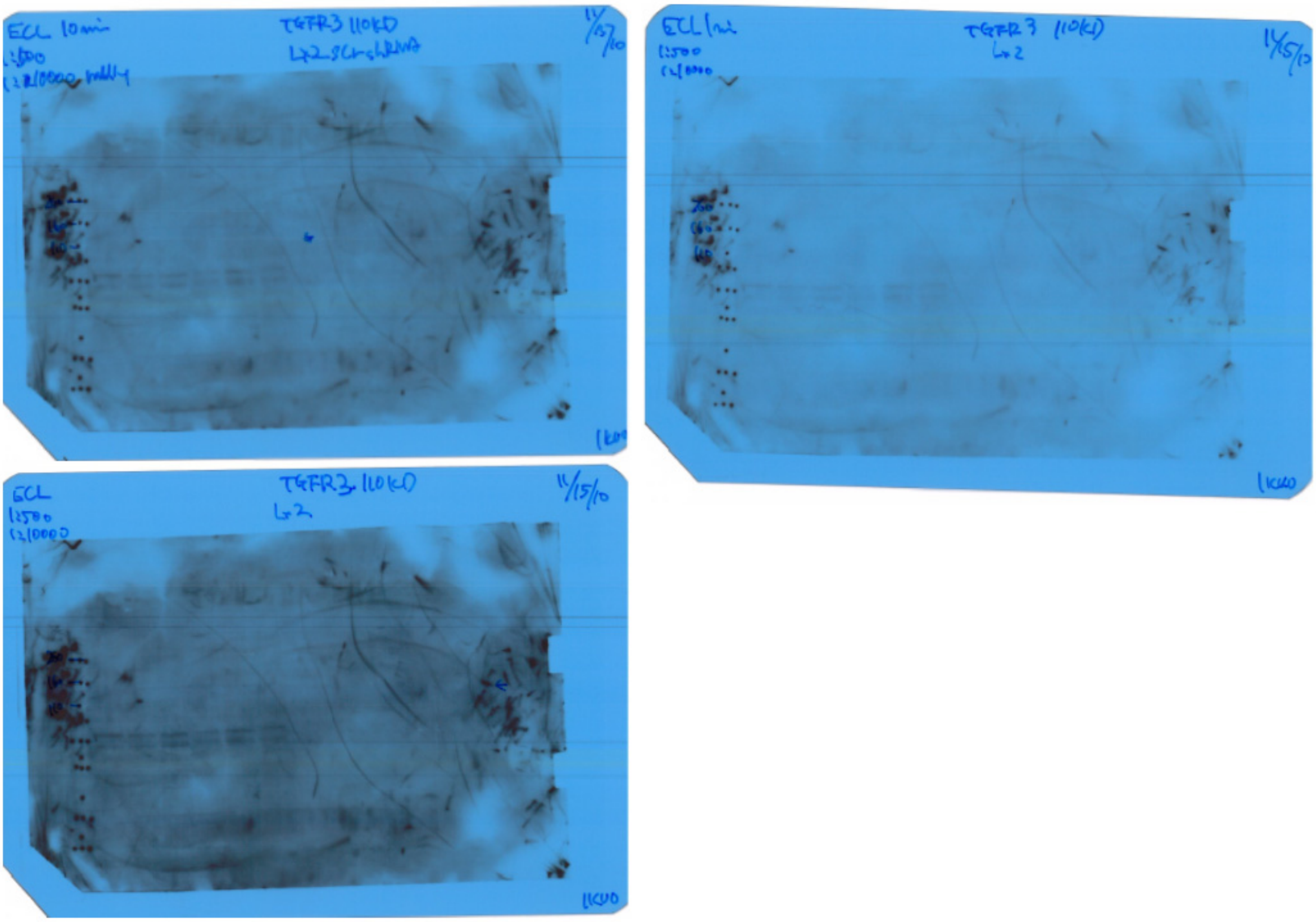
ECL 10min
TGFR3 110KD
ECL
TGFR3 (110KD)
ECL
TGFR3 110KD
11/15/10

(G)

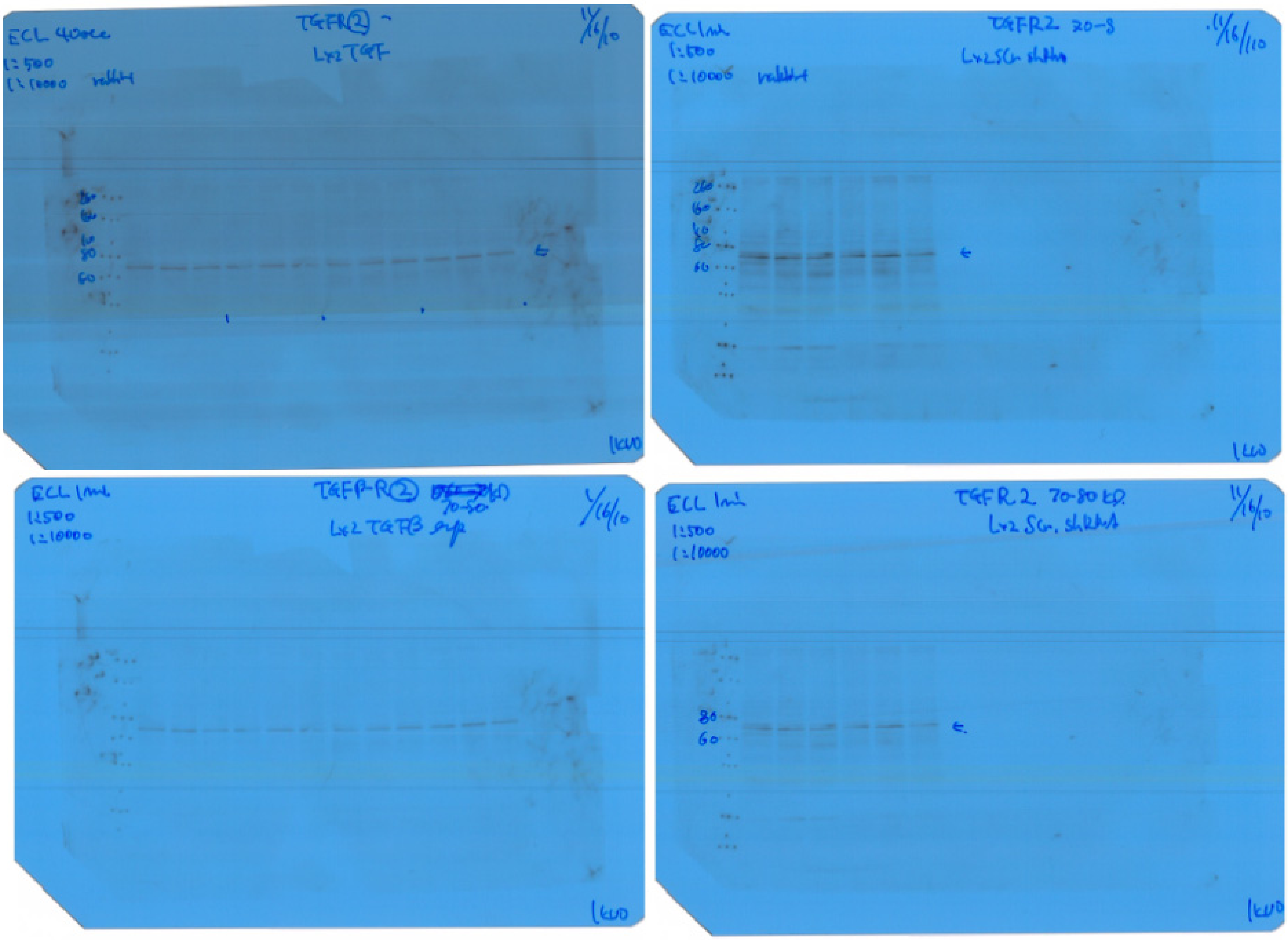

(**H**)

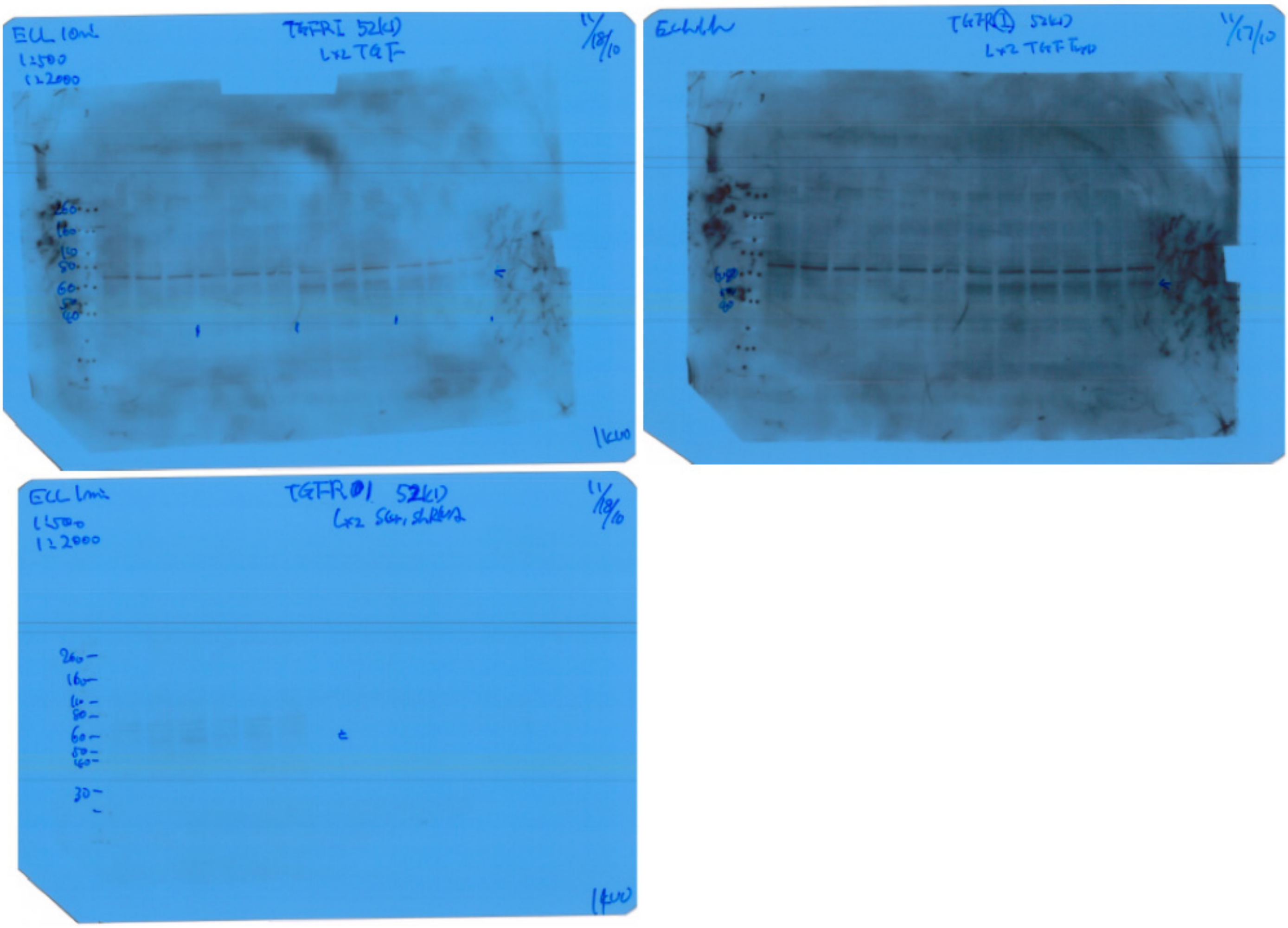

(I)

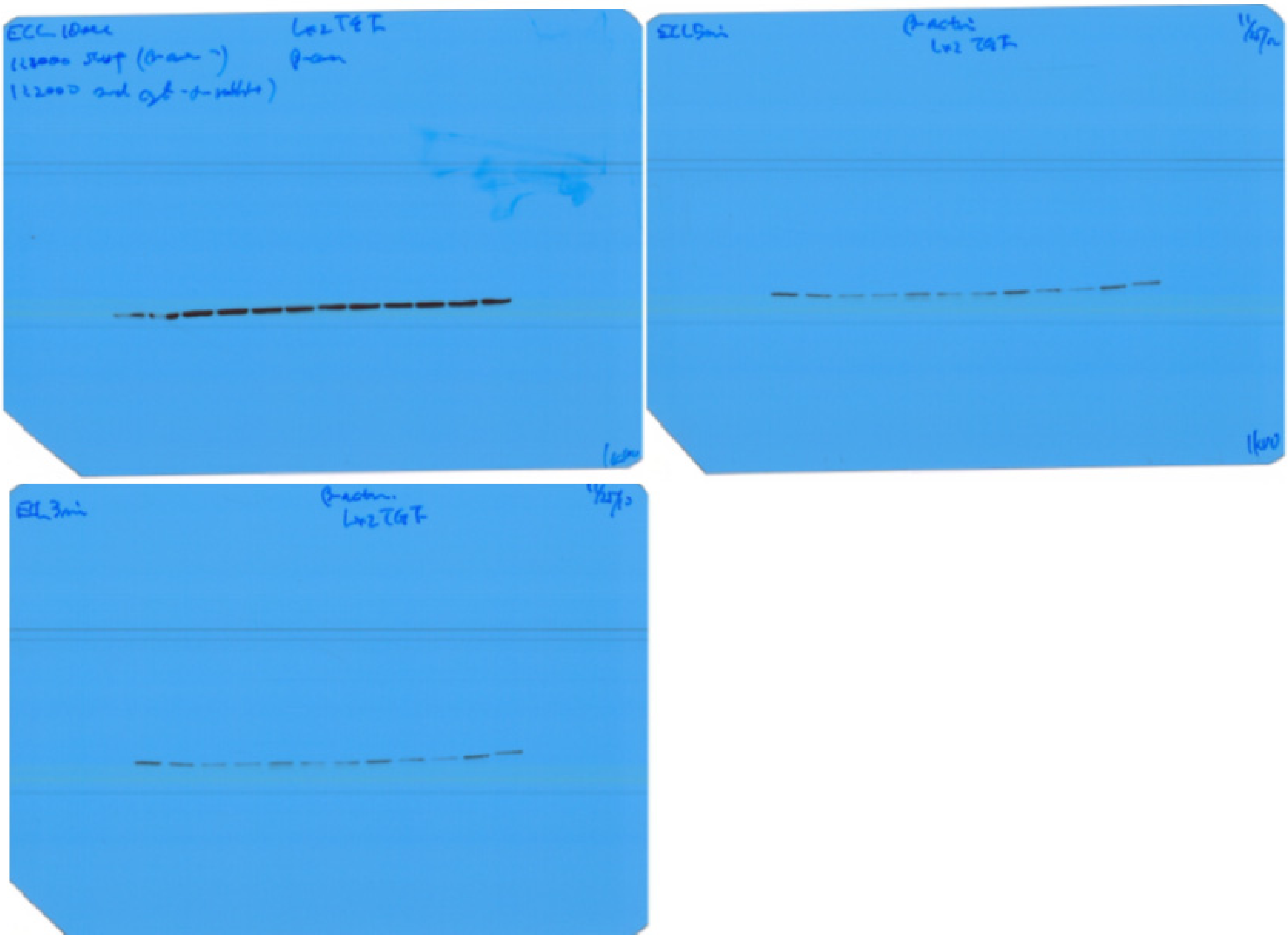

(J)

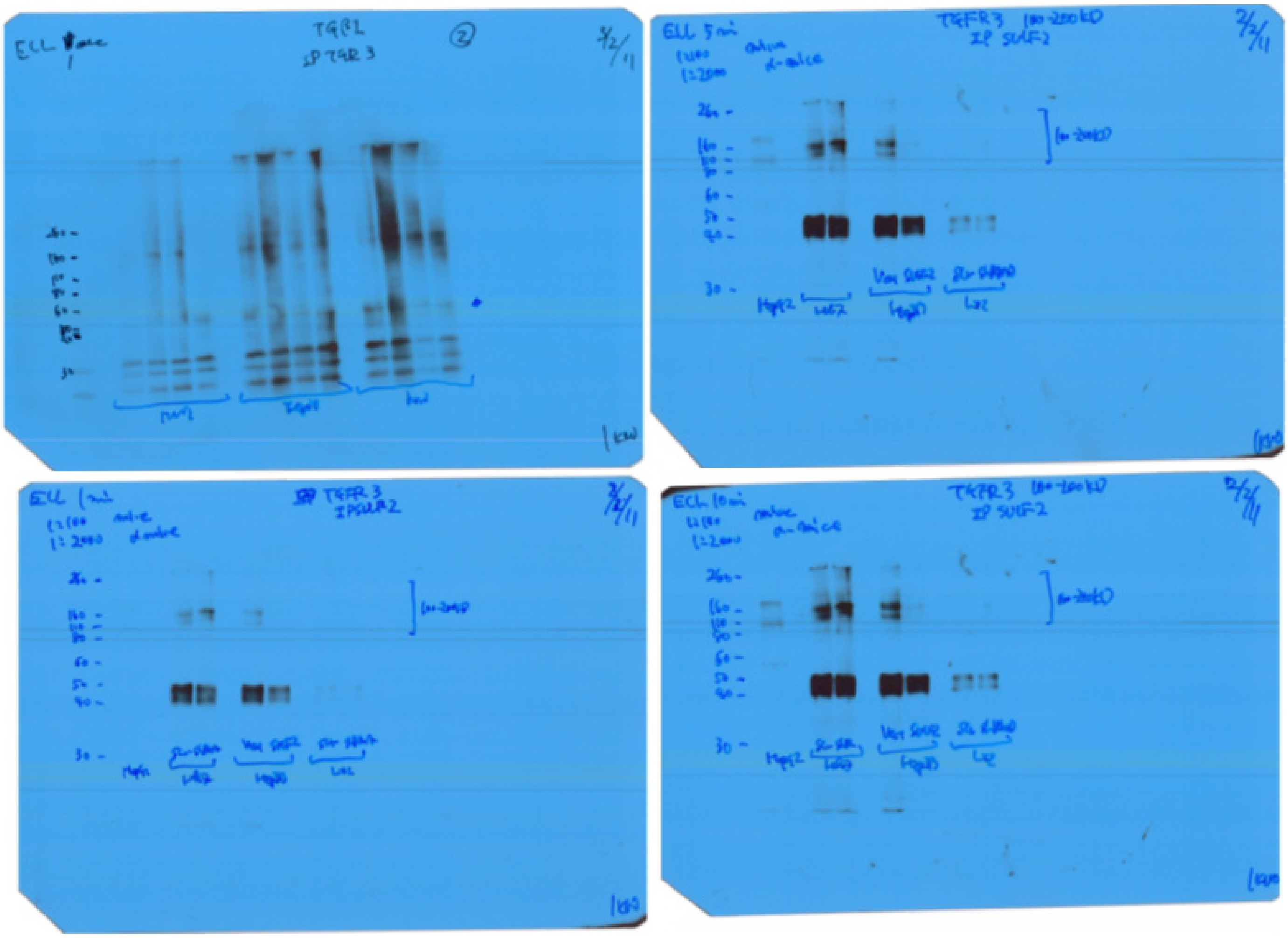

Figure S1. Uncropped Western Blot Figures for Figure 5 and Figure 7. (**A**) Figure 5. LX2 Cells. α-SMA; (**B**) Figure 5. LX2 Cells. Total SMAD3; (**C**) Figure 5. LX2 Cells. phospho-SMAD3; (**D**) Figure 5. LX2 Cells. Total SMAD2; (**E**) Figure 5: LX2 Cells. Phospho-SMAD2; (**F**) Figure 5. LX2 Cells. TGFBR3; (**G**) Figure 5. LX2 Cells. TGFBR2; (**H**) Figure 5. LX2 Cells. TGFBR1; (**I**) Figure 5. LX2 Cells. β-actin; (**J**) Figure 7. LX2 Cells. IP-TGFBR3/TGFBR1.

Table S1. Clinical and pathological features for individual liver samples.

Variables	Total (*n* = 45)	Non-Cirrhosis (*n* = 20)	Cirrhosis (*n* = 25)
Ages (Years)	64.2 (26–84)	60.3 (26–79)	67.0 (46–84)
Sex (Male/Female)	25/20	10/10	16/9
HBV-HCV		5	12
HCC		7	2
Alcohol		3	5
PBC		1	1
Autoimmune		2	2
Unknown		2	3
